# Supplementary material for: Phenotype of Mrps5-Associated Phylogenetic Polymorphisms Is Intimately Linked to Mitoribosomal Misreading
Source: Int J Mol Sci. 2022 Apr 15;23(8):4384. doi: 10.3390/ijms23084384 (PMC9030964; doi:10.3390/ijms23084384)
Supplement: Supplementary file 1 [file ijms-23-04384-s001.zip › ijms-1631061-supplementary.pdf]

Figure S1a

Alignment of bacterial RpsE (uS5), eukaryotic cytosolic Rps2 (uS5c), and mitochondrial Mrps5 (uS5m) ribosomal proteins

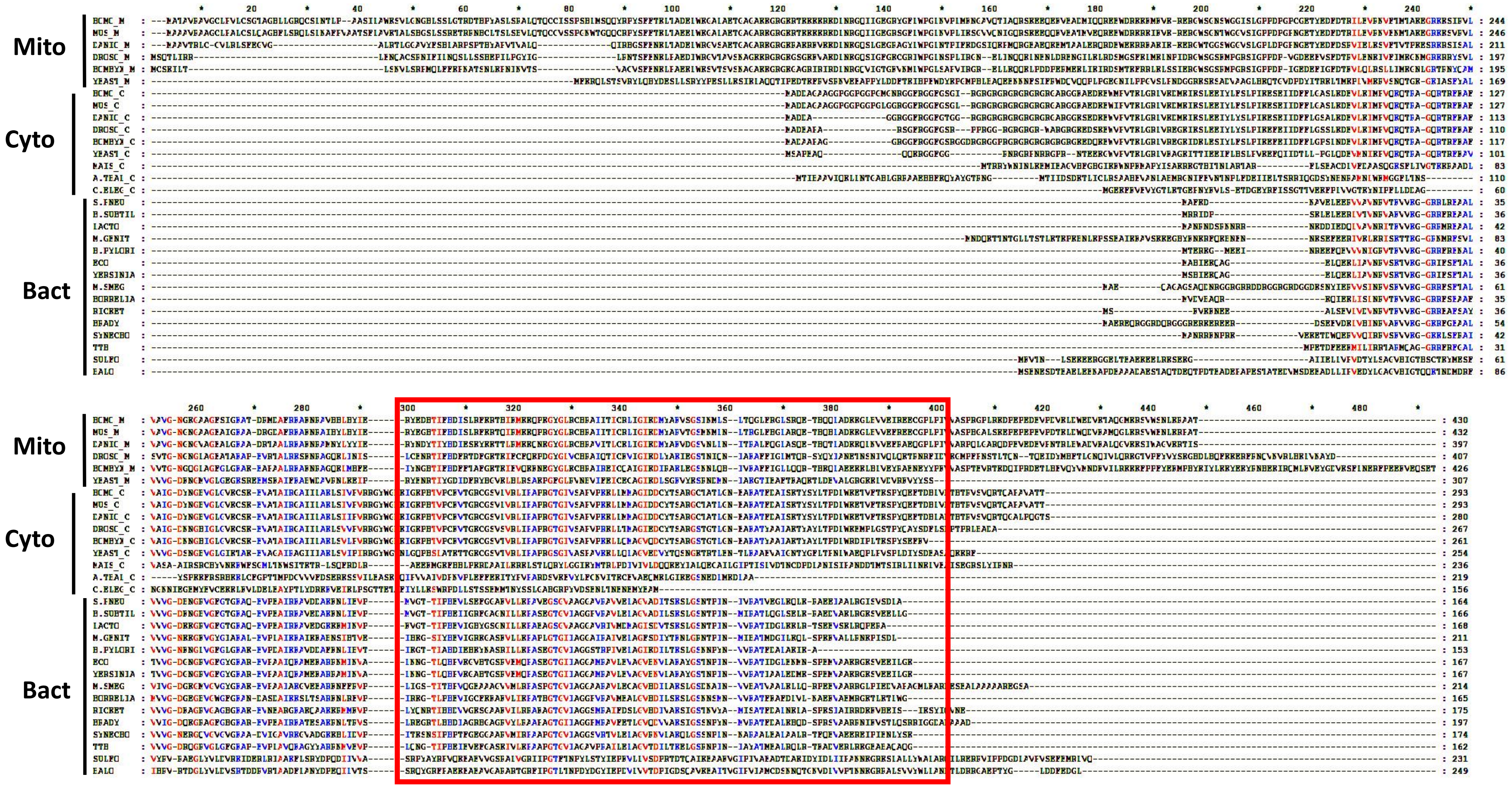

Part of the alignment inside the red frame is zoomed on the Figure S1b

Figure S1b

Alignment of bacterial RpsE (uS5), eukaryotic cytosolic Rps2 (uS5c), and mitochondrial Mrps5 (uS5m) ribosomal proteins, selected part

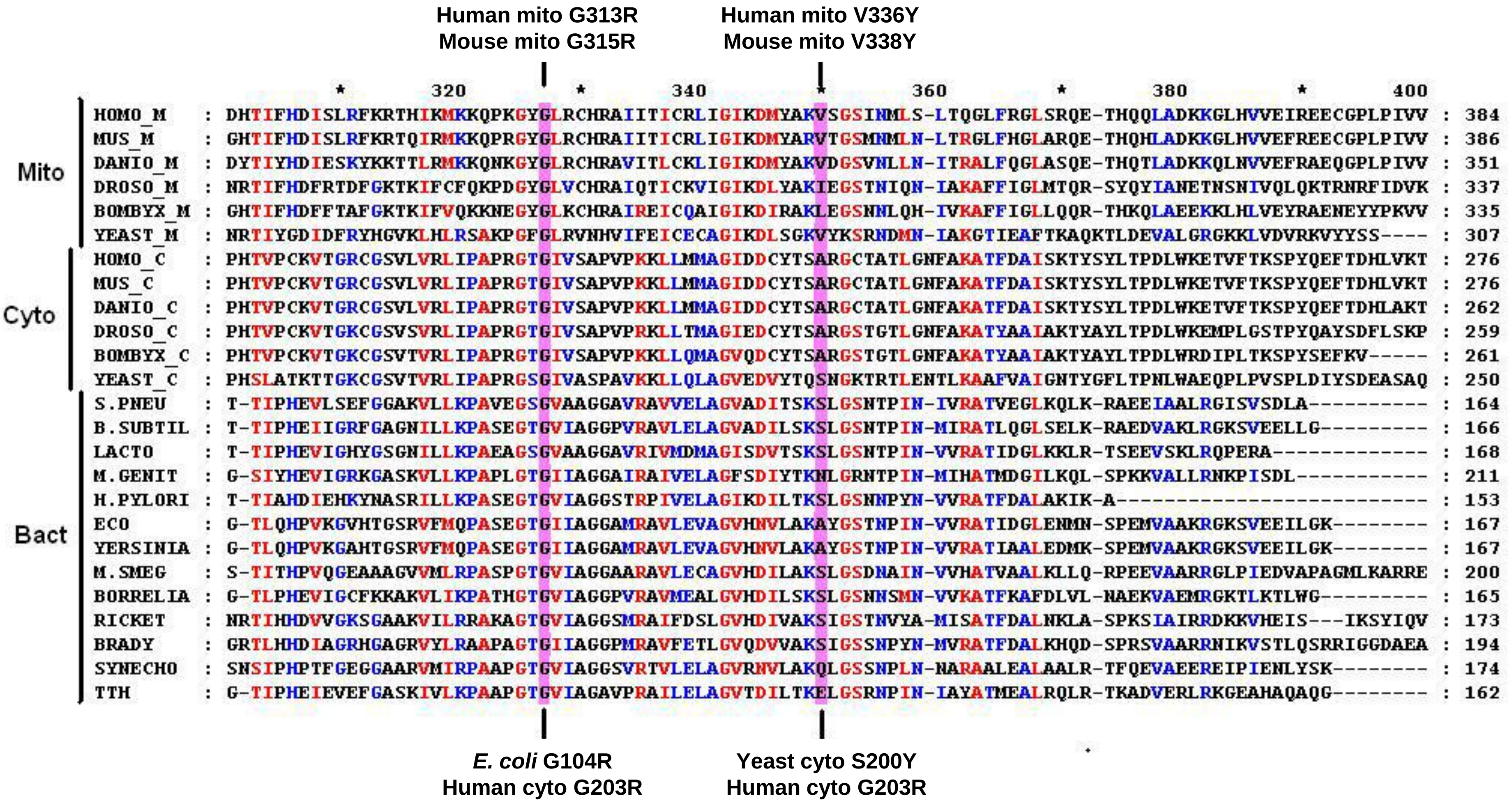

Figure S2

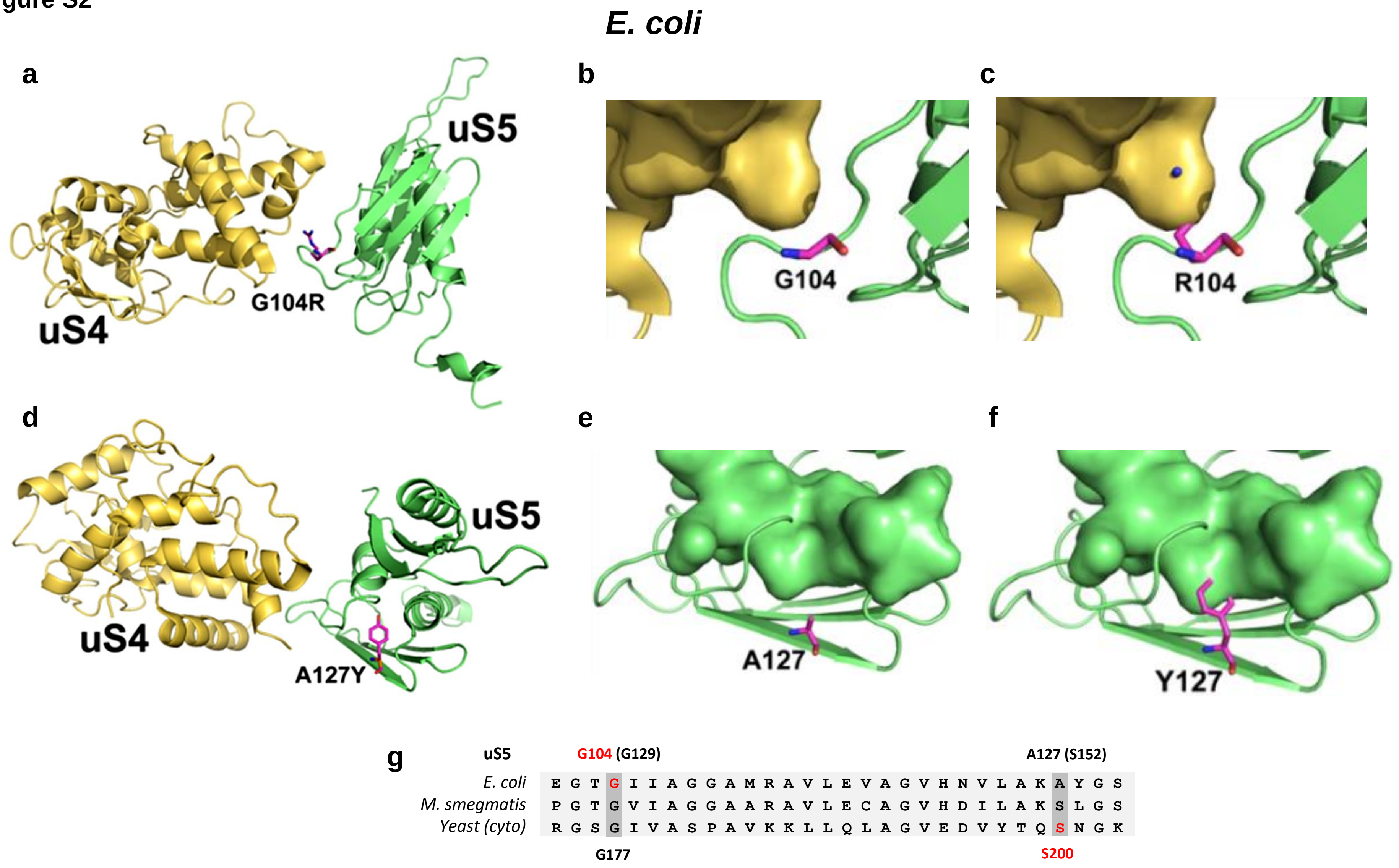

Figure S2. Modelling of amino acid substitutions in bacterial ribosomal protein RpsE (uS5). Visualization was done using PyMol v.1.8 (Schrödinger, Inc.) on the basis of published crystal structures. (**a**) Protein-protein interface uS4-uS5 in the *E. coli* ribosome. uS4 (RpsD) and uS5 (RpsE) are shown in gold and green, respectively. Residue G104 and mutation G104R are marked in magenta. (**b**) and (**c**) show steric hindrance induced by G104R mutation disturbing uS4-uS5 interface; for better visualization the α-helices are shown as surface (Source: PDB 4YBB). (**d**) Protein-protein interface uS4-uS5 in the *E. coli* ribosome (same as (**a**), but different perspective). Residue A127 and mutation A127Y are marked in magenta. (**e**) and (**f**) show steric hindrance within the C-terminal domain of uS5 for the A127Y mutation; for better visualization the α-helices are shown as surface (Source: PDB 4YBB). (**g**) Sequence alignment of *E. coli* and yeast ribosomal proteins uS5 (RpsE and Rps2). The described ram mutations are shown in red – *E.coli* numbering G104 and A127, yeast numbering G177 and S200.

Figure S3

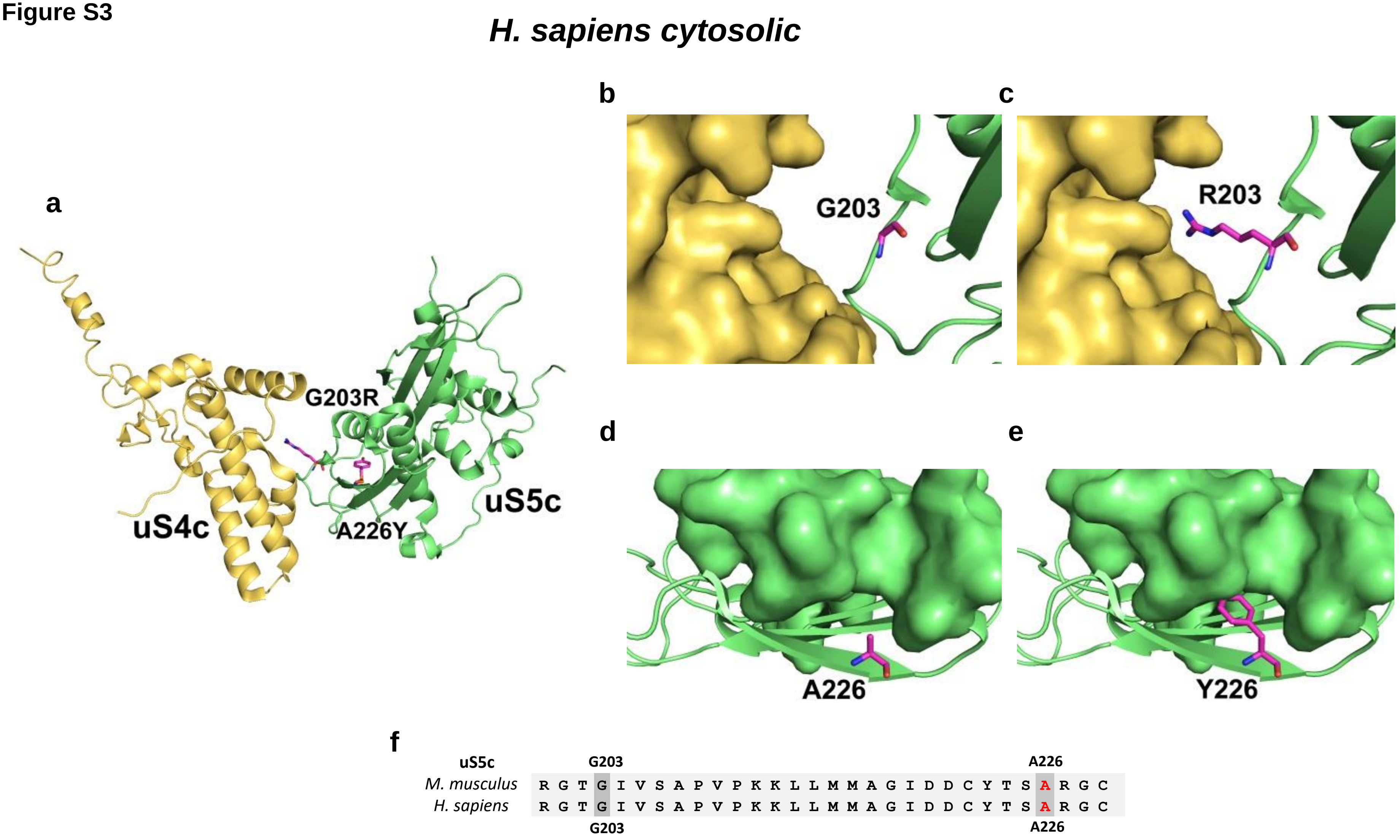

Figure S3 Modelling of amino acid substitutions in eukaryotic cytosolic ribosomal protein Rps2 (uS5c). Visualization was done using PyMol v.1.8 (Schrödinger, Inc.) on the basis of published crystal structures. (**a**) Protein-protein interface uS4c-uS5c in human cytosolic ribosome. **b**) and **c**) – absence of steric hindrance between uS4 and uS5 resulting from G203R mutation; to make it better visible the uS4c is shown as surface. **d**) and **e**) - steric hindrance within C-terminal domain of uS5c resulting from A226Y mutation. Source – PDB 4V6X. (**g**) Sequence alignment of *M. musculus* and *H. sapiens* uS5c. The mutated amino acid positions are indicated – G203 and A226 (mouse and human uS5c proteins share 100% identity). The described ram mutations are shown in red – A226Y.

Figure S4

H. sapiens mitochondrial

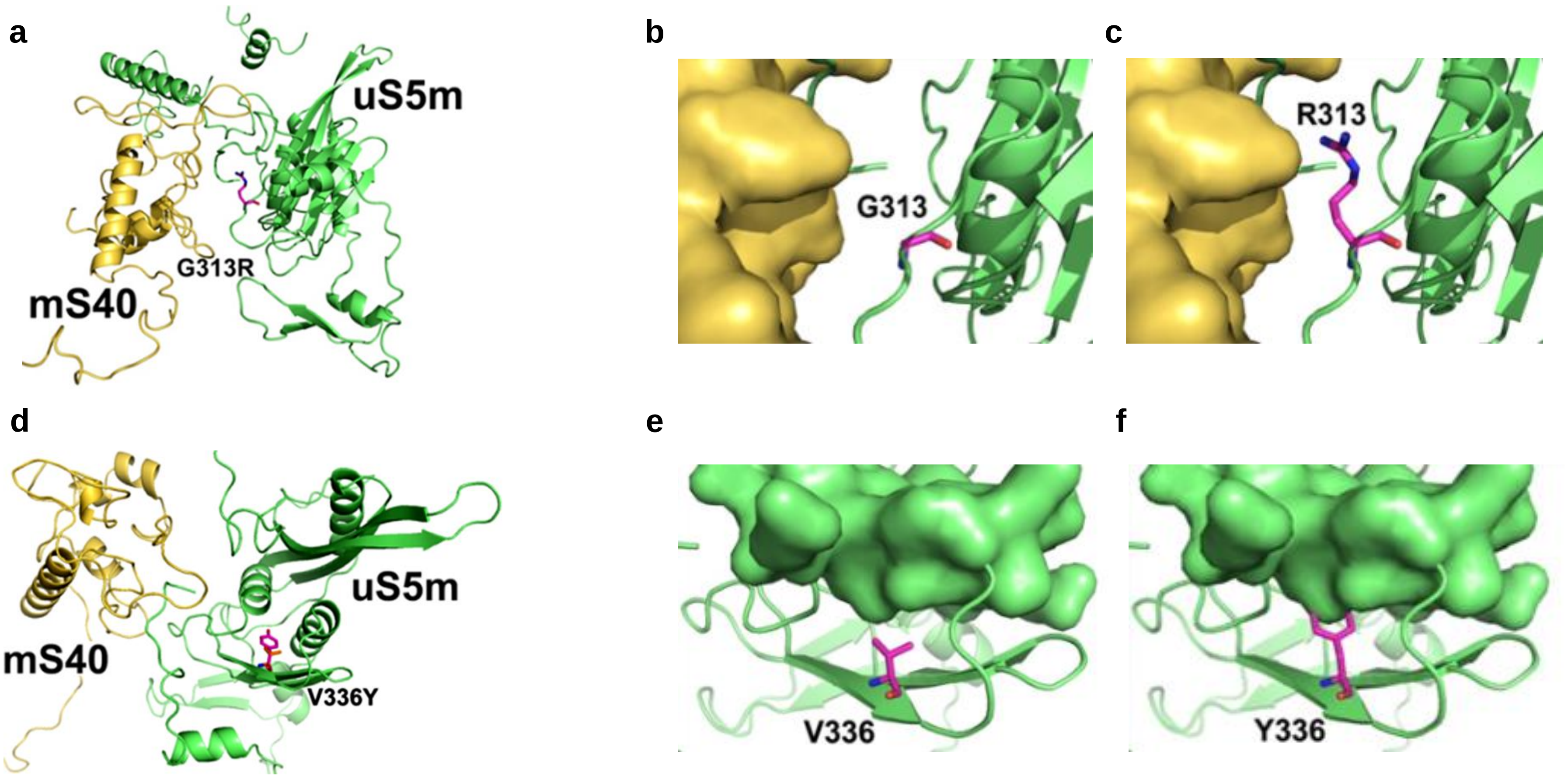

g

uS5m	G315 … V338
M. musculus	R G Y **G** L R C H R A I I T I C R L I G I K D M Y A R **V** T G S
H. sapiens	K G Y **G** L R C H R A I I T I C R L I G I K D M Y A K **V** S G S
	G313 … V336

Figure S4 Modelling of amino acid substitutions in eukaryotic mitochondrial ribosomal protein Mrps5 (uS5m). Visualization was done using PyMol v.1.8 (Schrödinger, Inc.) on the basis of published crystal structures. (**a**) Protein-protein interface mS40-uS5m in the human mitochondrial ribosome. Mitochondria-specific ribosomal protein mS40 structurally replaces uS4 in the mitoribosome. mS40 and uS5m are shown in gold and green, respectively. Residue G313 and mutation G313R are marked in magenta. (**b**) and (**c**) – lack of steric hindrance resulting from G313R mutation at the interface between mS40 and uS5m; for better visualization the α-helices are shown as surface (Source: PDB 3J9M). (**d**) Protein-protein interface mS40-uS5m in the human mitochondrial ribosome (same as (**a**), but different perspective). (**e**) and (**f**) – steric hindrance within C-terminal domain of uS5m resulting from V336Y mutation; for better visualization the α-helices are shown as surface (Source: PDB 3J9M). (**g**) Sequence alignment of *M. musculus* and *H. sapiens* uS5m. The mutated amino acid position is indicated – *M. musculus* numbering G315 and V338, *H. sapiens* numbering G313 and V336. The described ram mutations are shown in red – *M. musculus* V338Y and *H. sapiens* V336Y.

Figure S5

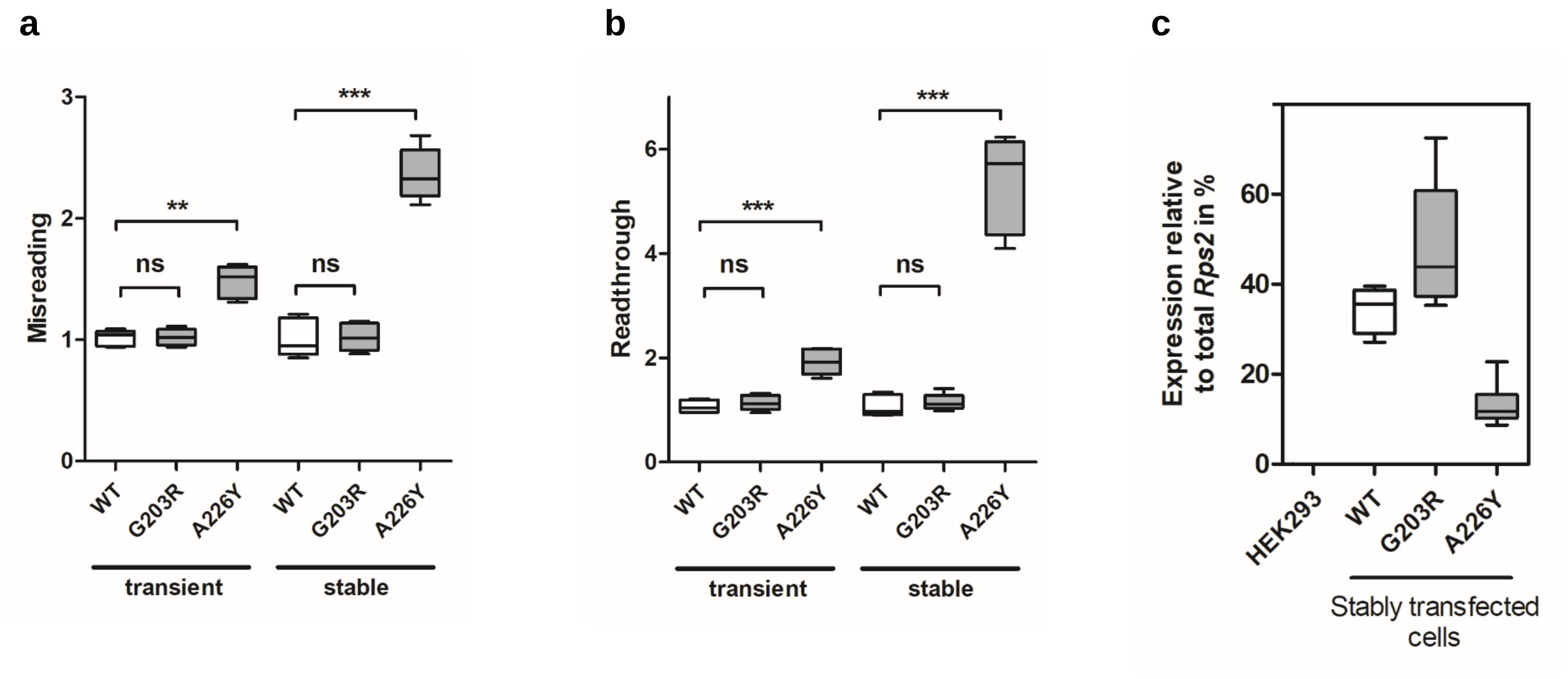

Figure S5. Characterization of the Rps2 G203R mutant protein. (**a**) Relative mRNA expression of *RPS2 G203R* transgene in comparison to endogenous *RPS2* in stably transfected HEK293 cells determined by qPCR (n=5 clones; whiskers: min-max). (**b**) Misreading and (**c**) readthrough in transiently or stably transfected cells measured by dual luciferase gain-of-function *in vivo* assays. Results are derived by calculating mutant hFluc/hRluc activity related to wild-type hFluc/hRluc activity, wild-type samples were set as 1. Whiskers: min-max; **P<0.01, ***P<0.001, n.s. - not significant.

Figure S6

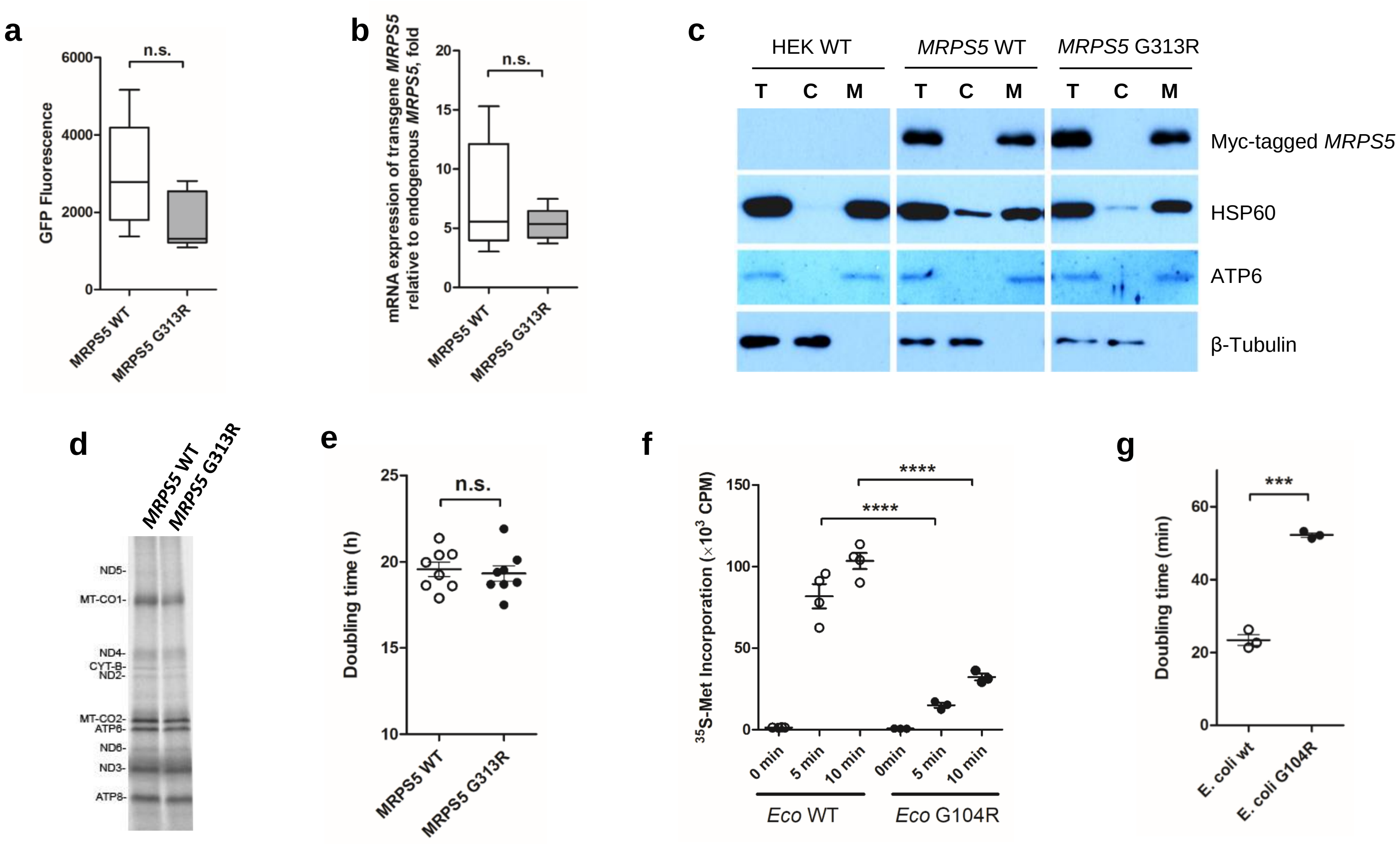

Figure S6. Characterization of the cells expressing Mrps5 G313R mutant protein or homologous RpsE G104R mutant protein. (**a**) GFP fluorescence of HEK cells stably transfected with *MRPS5* WT or *MRPS5* G313R (n=8 clones each, Whiskers: min-max, n.s., not significant). (**b**) Relative mRNA expression of *MRPS5* transgene in comparison to endogenous *MRPS5* in stably transfected HEK293 cells determined by qPCR (n=8 clones, Whiskers: min-max, n.s., not significant). (**c**) Western Blot showing cellular localization of myc-tagged MrpS5 protein. HEK293 cells were transiently transfected using *MRPS5* wild-type or *MRPS5* G313R constructs. Cell lysates were fractionated (T - total, C – cytosolic fraction, M - mitochondrial fraction) and Western Blot was performed. Myc-tag antibody (Ab) was used to detect MrpS5; Hsp60 served as a marker for proteins produced in the cytosol and localized in mitochondria; ATP6 served as a marker for proteins synthesized and localized in mitochondria, β-tubulin served as a marker for proteins translated and localized in the cytosol. (**d**) Autoradiography of SDS PAGE analysis of the *in organello* translation (detection with 35S-Met labelling). (**e**) Doubling time of the MRPS5 WT and MRPS5 G313R transfected cells (n=8 clones for each). Whiskers: min-max; error bars ±SEM. (**f**) Translation efficiency in *E. coli* wild-type and G104R mutant strains assessed by ^{35}S-Met incorporation (n=4, ±SEM, ****P<0.0001). (**g**) Generation time of *E.coli* wild-type and G104R mutant strains (n=3, ±SEM, ***P<0.001).

Figure S7

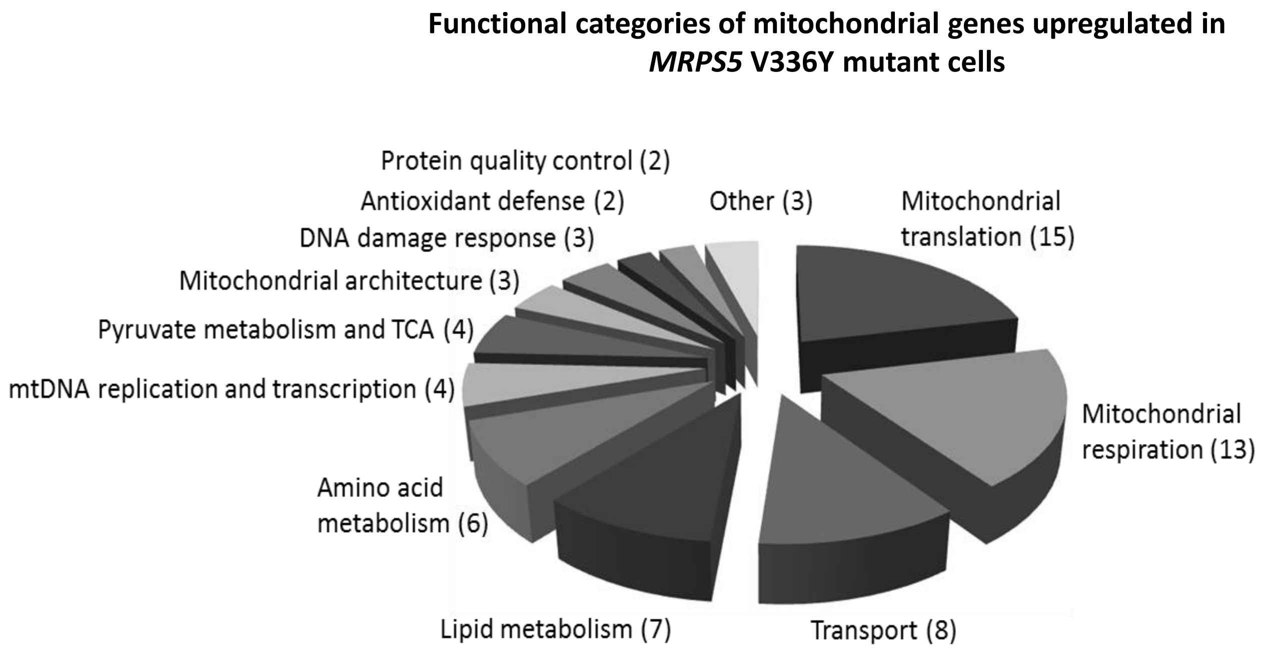

Figure S7. Transcriptome profiling analysis of cells stably expressing with Mrps5 V336Y mutant protein compared to wild-type control. Functional grouping of mitochondrial genes upregulated in MRPS5 V336Y mutants (derived from terms “mitochondrial ribosome”, “mitochondrial matrix”, “mitochondrial respiratory chain complex I”, and “mitochondrial inner membrane” enriched in GO Cellular Component analysis) according to their known or suggested functions. The segment sizes correspond to the number of genes in each category related to the total number of upregulated mitochondrial genes. Numbers in brackets refer to the number of genes in each category.

Figure S8

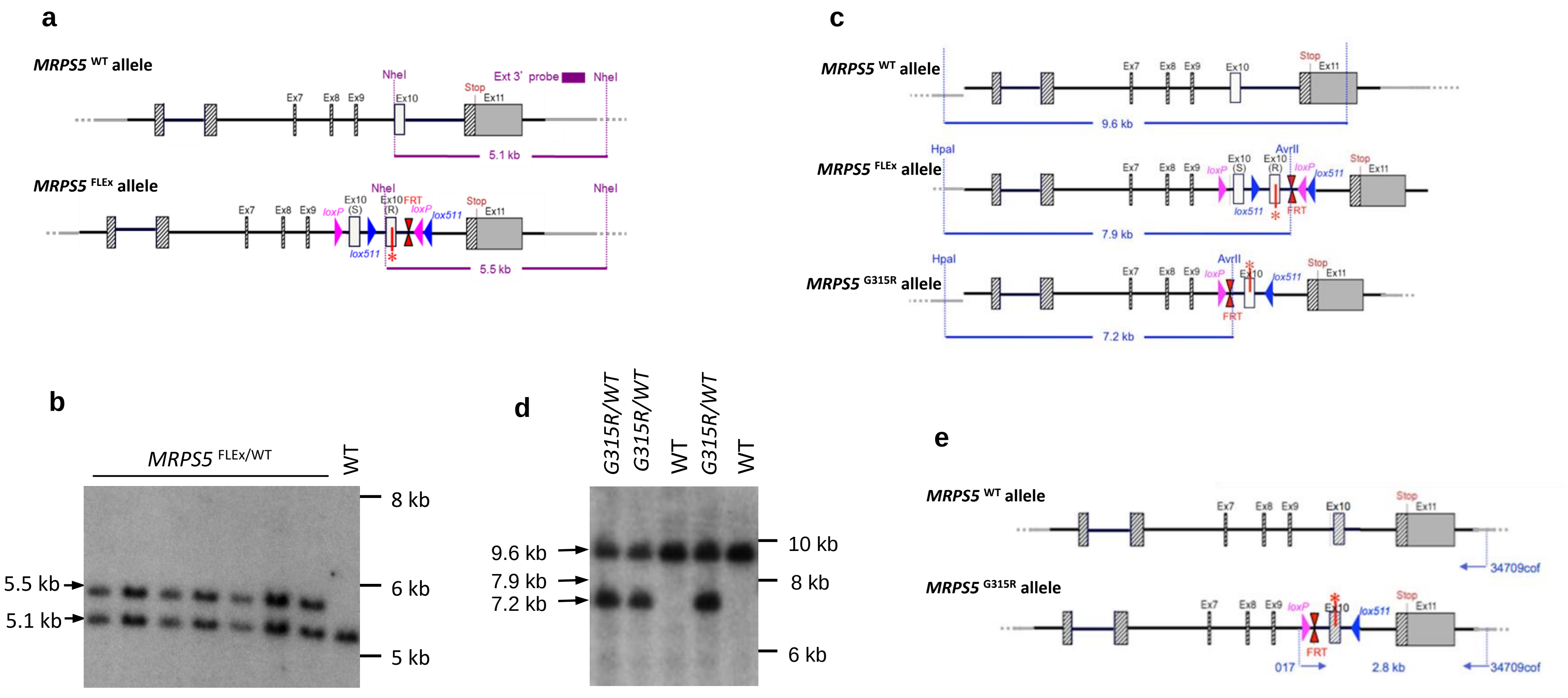

Figure S8. Generation of *MRPS5*$^{G315R/G315R}$ mutant mice. (**a**) Southern blot strategy for detection of *MRPS5*^{FLEx} allele. Schematic representation of *MRPS5* wild-type allele and inducible mutant *MRPS5* Knock-in (*MRPS5*^{FLEx}) allele. Restriction sites used are indicated. (**b**) Representative example of Southern blot analysis. Southern blot was performed using double NheI digestion of genomic DNA and hybridization with the 3' probe indicated. (**c**) Southern blot strategy for detection of heterozygous induced mutant *MRPS5*$^{G315R/WT}$ mice. Schematic representation of *MRPS5* wild-type, *MRPS5*^{FLEx}, and induced mutant *MRPS5* Knock-in (*MRPS5*^{G315R}) allele. Restriction sites used are indicated. (**d**) Representative example of Southern blot genotyping. Southern blot was performed using double AvrII/HpaI digestion of genomic DNA and hybridization with the external 5' probe indicated to detect inducible or induced mutant *MRPS5* allele and *MRPS5* wild-type allele. Wild-type controls are indicated (WT). (**e**) Detection of the G315R point mutation in the induced mutant *MRPS5*$^{G315R/WT}$ Knock-in mice. Schematic representation of the PCR strategy, primers are specific for the induced mutant *MRPS5*^{G315R} allele.

Figure S8 (continued)

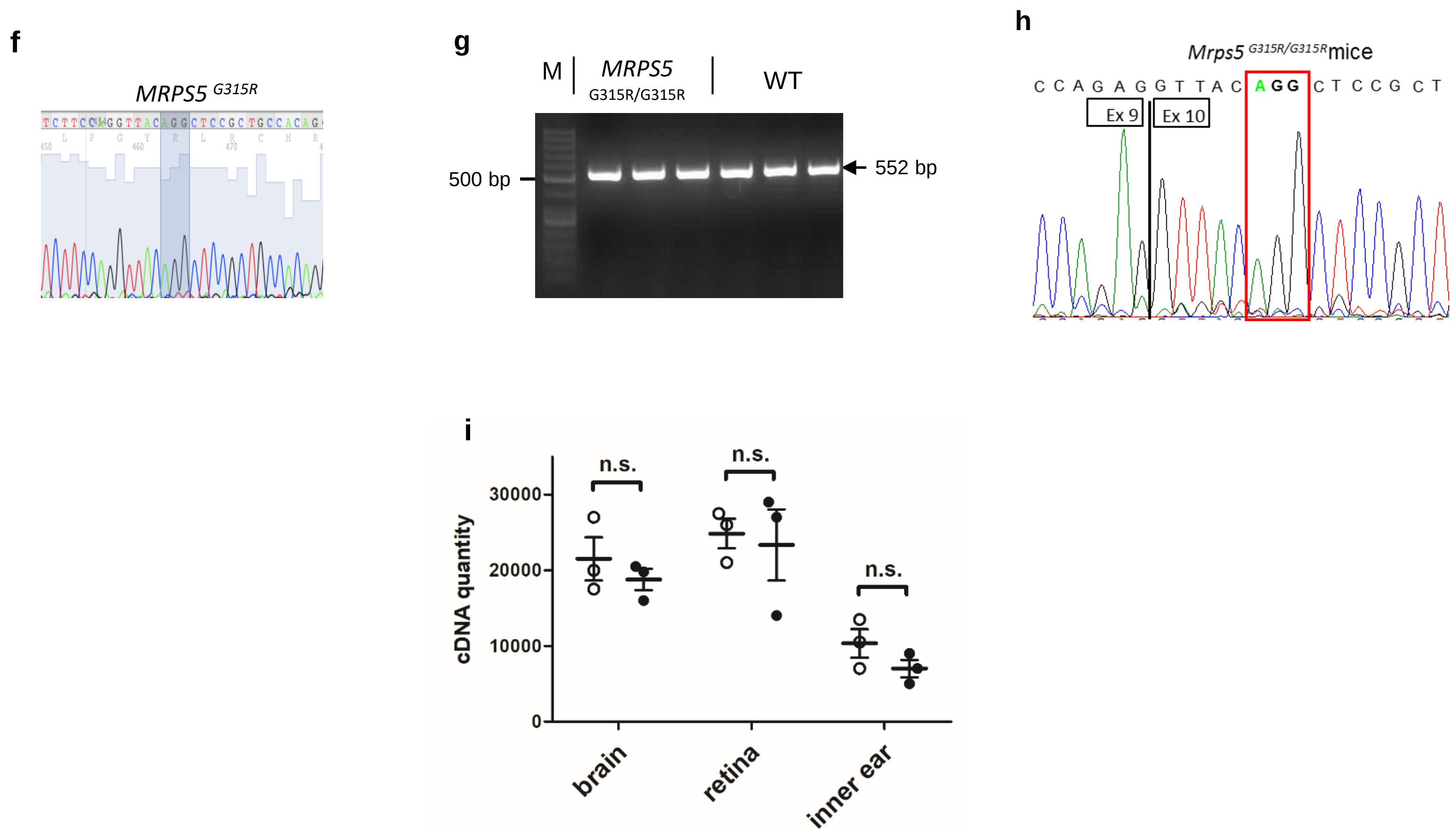

Figure S8 (continued). (**f**) Representative DNA sequencing result of *MRPS5*$^{G315R/G315R}$ homozygous mice. (**g**) Representative RT-PCR result of *MRPS5*$^{G315R/G315R}$ and wild-type mice. (**h**) Representative sequencing result of *MRPS5*$^{G315R/G315R}$ RT-PCR amplicon. (**i**) Quantification of *MRPS5* transcripts in brain, inner ear and retina from *MRPS5*$^{G315R/G315R}$ mice and wild-type mice. Mean values of cDNA quantity for wild-type (○) and *MRPS5*$^{G315R/G315R}$ (●) mice are shown, error bars represent SEM.

Figure S9

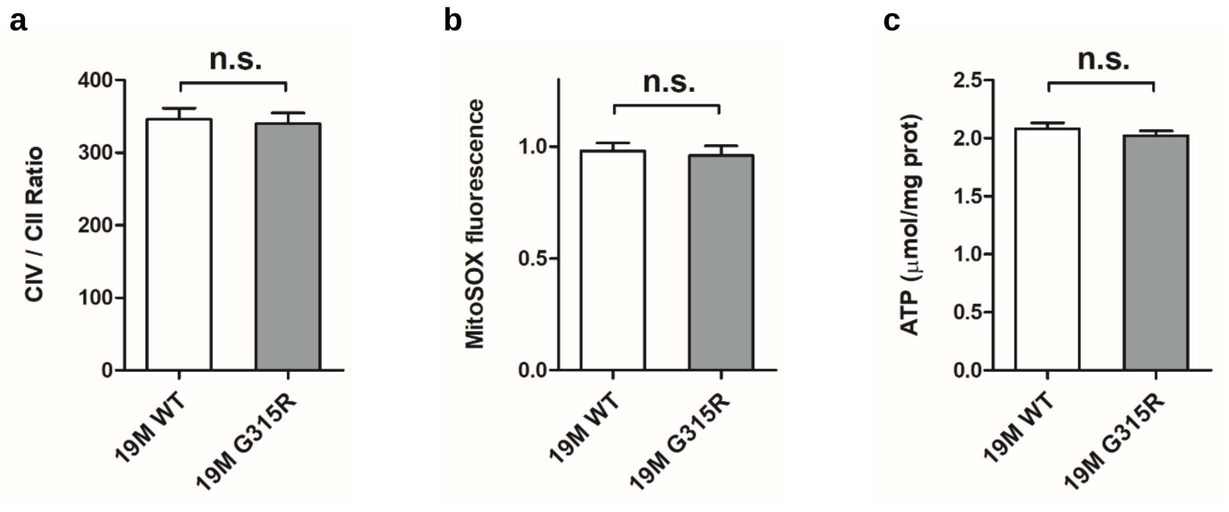

Figure S9. Effect of the mutation MRPS5 G315R on mitochondrial function in the brain of 19 months old mice. (**a**) Ratio of mitochondrial complex IV and complex II activities (n=3 replicates of 5 animals, ±SEM). (**b**) Superoxide anion radicals detected by MitoSOX fluorescence (n=3 replicates of 5 animals, ±SEM). (**c**) ATP concentrations adjusted to total protein content (n=5 replicates of 5 animals, ±SEM).

Figure S10

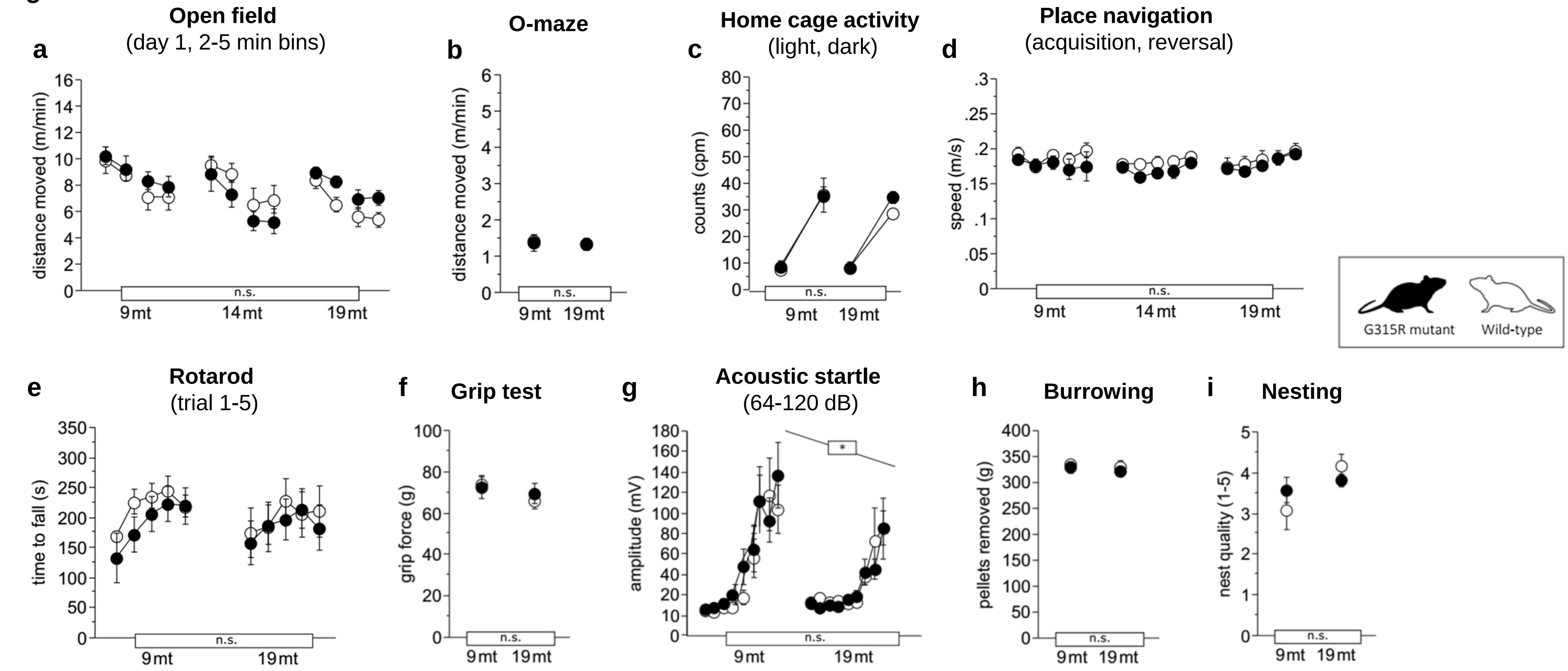

Figure S10. Activity in familiar and novel environments (a-c), sensory-motor function (d-g) and species-typical behaviors (h-i). (**a**) Large open field, distance moved (normalized to 1 min observation time) (ANOVA: genotype $F_{1,34}=0.321$ ns, age $F_{2,34}=2.521$ p=.0953, time $F_{3,102}=59.12$ p<.0001, age x genotype $F_{2,34}=1.805$ ns, time x genotype $F_{3,102}=0.210$ ns, time x age $F_{6,102}=1.092$ ns). (**b**) Elevated O-maze, distance moved (normalized to 1 min observation time) (ANOVA: genotype $F_{1,35}=0.287$ ns, age $F_{2,35}=0.137$ ns, age x genotype $F_{2,35}=0.162$ ns). (**c**) Spontaneous activity in the individual home cage monitored by IR sensors, counts per minute during the light and dark phase averaged over 6 days (ANOVA: genotype $F_{1,36}=0.163$ ns, age $F_{2,36}=0.481$ ns, phase $F_{1,36}=658.8$ p<.0001, age x genotype $F_{2,36}=0.414$ ns, phase x genotype $F_{1,36}=1.374$ ns, phase x age $F_{2,36}=1.191$ ns). (**d**) Place navigation task, average daily swim speed during acquisition and reversal (ANOVA: genotype $F_{1,33}=1.720$ ns, age $F_{2,33}=1.866$ ns, time $F_{4,132}=10.33$ p<.0001, age x genotype $F_{2,33}=0.356$ ns, time x genotype $F_{4,132}=0.152$ ns, time x age $F_{8,132}=1.455$ ns). (**e**) Accelerating rotarod, time to fall during trial 1-5 (ANOVA: genotype $F_{1,34}=1.262$ ns, age $F_{2,34}=0.103$ ns, trial $F_{4,136}=8.960$ p<.0001, age x genotype $F_{2,34}=0.071$ ns, trial x genotype $F_{4,136}=0.230$ ns, trial x age $F_{8,136}=0.439$ ns). (**f**) Grip force, average of 10 trials (ANOVA: genotype $F_{1,36}=0.343$ ns, age $F_{2,36}=9.752$ p=.0004, age x genotype $F_{2,36}=0.119$ ns). (**g**) Acoustic startle profile, max. response amplitude to stimuli of 64, 68, 72, 76, 80, 90, 100, 110, 120 dB (ANOVA: genotype $F_{1,34}=0.267$ ns, age $F_{2,34}=3.364$ p=.0464, stimulus $F_{2,68}=6.629$ p=.0023, age x genotype $F_{2,34}=0.050$ ns, stimulus x genotype $F_{2,68}=1.471$ ns, stimulus x age $F_{4,68}=1.090$ ns). (**h**) Burrowing test, pellets removed after 24h (ANOVA: genotype $F_{1,34}=0.257$ ns, age $F_{2,34}=5.607$ p=.0079, age x genotype $F_{2,34}=0.324$ ns). (**i**) Nesting test, next quality score after 24h (ANOVA: genotype $F_{1,35}=0.012$ ns, age $F_{2,35}=1.820$ ns, age x genotype $F_{2,35}=0.926$ ns). Graphs show mean and SE. Boxes on the x-axis indicate significance of genotype, boxes above data points indicate significance of age, *P<0.05, n.s. P≥0.05. Mutants are depicted with black circles; wild-types with white circles. 34 mice, n=17 per genotype, n=9-13 per age cohort.
